# The association between perihaematomal oedema and functional outcome after spontaneous intracerebral haemorrhage: A systematic review and meta-analysis

**DOI:** 10.1177/23969873231157884

**Published:** 2023-02-18

**Authors:** Maaike P Cliteur, Lotte Sondag, Laura Cunningham, Rustam Al-Shahi Salman, Neshika Samarasekera, Catharina JM Klijn, Floris HBM Schreuder

**Affiliations:** 1 Department of Neurology, Donders Institute for Brain, Cognition and Behaviour, Radboud University Medical Centre, Nijmegen, The Netherlands; 2 Centre for Clinical Brain Sciences, The University of Edinburgh, Edinburgh, UK

**Keywords:** Intracerebral haemorrhage, perihaematomal oedema, systematic review, meta-analysis

## Abstract

**Purpose:**

Perihaematomal oedema (PHO) formation has gained increasing interest as a therapeutic target after spontaneous intracerebral haemorrhage (ICH). Whether PHO contributes to poor outcome is unclear. We aimed to determine the association between PHO and outcome in patients with spontaneous ICH.

**Method:**

We searched five databases up to 17 November 2021 for studies of ⩾10 adults with ICH reporting the presence of PHO and outcome. We assessed risk of bias, extracted aggregate data and used random effects meta-analysis to pool studies that reported odds ratios (OR) with 95% confidence intervals (CI). Primary outcome was poor functional outcome defined as modified Rankin Scale score of 3–6 at 3 months. Additionally, we assessed PHO growth and poor outcome at any time of follow-up. We prospectively registered the protocol in PROSPERO (CRD42020157088).

**Findings:**

We identified 12,968 articles, of which we included 27 studies (*n* = 9534). Eighteen studies reported an association between larger PHO volume and poor outcome, six a neutral result and three an inverse relationship. Larger absolute PHO volume was associated with poor functional outcome at 3 months (OR per mL increase of absolute PHO 1.03, 95% CI 1.00–1.06, *I*^2^ 44%, four studies). Additionally, PHO growth was associated with poor outcome (OR 1.04, 95% CI 1.02–1.06, *I*^2^ 0%, seven studies).

**Discussion:**

In patients with spontaneous ICH, larger PHO volume is associated with poor functional outcome at 3 months. These findings support the development and investigation of new therapeutic interventions targeting PHO formation to evaluate if reduction of PHO improves outcome after ICH.

## Introduction

Spontaneous intracerebral haemorrhage (ICH) is the second most common subtype of stroke, affecting more than 3 million people worldwide each year.^1^ One-month case fatality rate is approximately 40%.^2^ Apart from the effect of stroke unit care and early control of elevated blood pressure that may be beneficial, there are no treatments with proven benefit.^3,4^

The mechanisms leading to brain injury in ICH are complex and can be divided into two main categories.^5^ Primary brain injury results from the immediate disruptive mass effect caused by the haematoma and occurs within the first hours after ICH.^5^ Subsequently, the local tissue damage stimulates the release of inflammatory factors, blood-brain-barrier breakdown, the activation of microglia and influx of circulating inflammatory cells.^6^ These secondary processes result in secondary brain injury and the development of perihaematomal oedema (PHO). Development of PHO may be detrimental via enhancement of the harmful mass effect but toxic dysregulation of the local osmotic gradient has also been suggested.^7^ PHO is considered a quantifiable radiological marker of secondary brain injury^8^ and has been used as an outcome measure in previous clinical studies targeting secondary brain injury after ICH.^9,10^ However, whether PHO affects clinical outcome after spontaneous ICH remains controversial as previous reports have shown conflicting results.^8,11,12^

We aimed to systematically review the literature and meta-analyse studies that investigated the association between PHO and outcome in adults with spontaneous ICH.

## Methods

### Search strategy and study selection

We searched MEDLINE, Embase, Cochrane library, clinicaltrials.gov and International Standard Randomised Controlled Trial Number Register (ISRCTN) up to 17 November 2021 for published prospective or retrospective observational cohort studies, case control studies and randomised controlled trials in human adults that investigated the association between PHO and outcome ⩾30 days after symptom onset. We performed an electronic search strategy consisting of different combinations of the terms for ICH AND (perihaematomal) oedema (Supplemental Material). There were no restrictions on language or publication date. Studies had to comprise at least 10 patients to be included. PHO could be reported as absolute PHO volume (aPHO), relative PHO volume (rPHO = $\frac{\mathrm{aPHO}}{\mathrm{ICH}\;\mathrm{volume}}$ ) or oedema extension distance (OED = $\sqrt[{3}]{\frac{PHO\;volume+ICH\;volume}{\frac{4}{3}\pi }}-\sqrt[{3}]{\frac{ICH\;volume}{\frac{4}{3}\pi }}$ ).^13^ Imaging modalities to measure PHO could be computed tomography (CT) or magnetic resonance imaging (MRI). The following studies were excluded: conference abstracts, studies regarding ICH secondary to an underlying macrovascular cause identified by brain imaging, studies including solely children (<18 years) and studies that reported in-hospital outcome measures only, because outcome of patients with ICH can improve after (longer) time.^14^ The study protocol was prospectively registered with the International Prospective Register of Systematic Reviews (CRD42020157088).

All records of potentially eligible studies were imported into Covidence (covidence.org). Two of four authors (MC, LS, FS, NS) independently screened all abstracts and two authors (MC, LS) assessed full texts to identify studies that met the predefined inclusion criteria. Disagreements were resolved by a third author (FS or NS). When two studies used overlapping cohorts, the study with the largest number of patients that best matched the inclusion criteria was included in our primary analysis. This process was repeated for the secondary analyses.

### Data extraction

Two authors (LS and MC) independently performed the methodologic quality assessment of the included studies using the Newcastle-Ottawa Quality scale (NOS) for cohort studies and for case control studies, with 0 points reflecting the highest risk of bias and 9 reflecting the lowest risk of bias. Studies scoring 0–3 points were considered of poor quality, with 4–6 points reflecting fair quality and 7–9 points good quality. Using a prespecified structured data extraction form, two authors (LS and MC) extracted the following data from all included studies: first author, year of publication, in- and exclusion criteria, baseline characteristics of the included subjects (age, sex, blood pressure on admission, medical history, medication use), ICH imaging characteristics (imaging modality, location, ICH volume, time since symptom onset), PHO parameters (aPHO, rPHO, OED, PHO growth, modality and timing of imaging), the number of patients with a certain functional outcome, the used definition of functional outcome and the timing of follow-up. We extracted this information for patients with good and poor outcome separately, to exclude patients without follow up. Discrepancies were resolved by discussion and, if necessary, by a third reviewer (FS) in a consensus meeting.

### Outcomes

The primary outcome was poor functional outcome at 3-month follow-up, defined as a dichotomised modified Rankin Scale (mRS) score of 3–6. Secondary outcomes were a mRS score 3–6 at any time of follow-up, a mRS score 4–6 at 3 months and death at any time of follow-up. For the primary and secondary analyses, we combined all studies that measured PHO by means of aPHO, rPHO and OED at any timepoint.

### Data analysis

We reported the effects of PHO on outcome in all included studies descriptively. We pooled reported odds ratio’s (ORs) with 95% confidence intervals (CI) for primary and secondary outcomes in a generic inverse-variance based random-effects method meta-analysis. When both unadjusted and adjusted ORs were available we included only the adjusted OR in the meta-analysis. Additionally, the association between PHO growth and any kind of reported outcome at any time of follow-up was analysed.

To maximise the number of studies contributing to our analyses, we also calculated standardised mean differences (SMD) between patients with a poor and patients with a good outcome for studies reporting crude aPHO measures for each outcome group, to determine whether results would support the results of the pooled OR for the primary outcome. When studies provided median and interquartile values (IQR), we approximated the sample mean and standard deviation (SD) following a standard method.^15^ Meta-analysis of SMDs was performed using a random effects model.

We assessed heterogeneity with the I-squared statistic (*I*^2^) and categorised heterogeneity as follows: 0%–40% heterogeneity that might not be important; 30%–60%, moderate heterogeneity; 50%–90%, substantial heterogeneity; and 75%–100%, considerable heterogeneity.

We aimed to investigate the following prespecified potential modifying factors by means of a meta-regression analysis if at least 10 studies in a meta-analysis had these data available: age, (systolic) blood pressure, the use of antithrombotic/antiplatelet agents or the use of statins. We constructed funnel plots to assess potential publication bias.

We used R and R-studio version 3.6.2 with packages ‘rmeta’ and ‘metafor’ for all statistical analysis.

## Results

**Figure 1.**
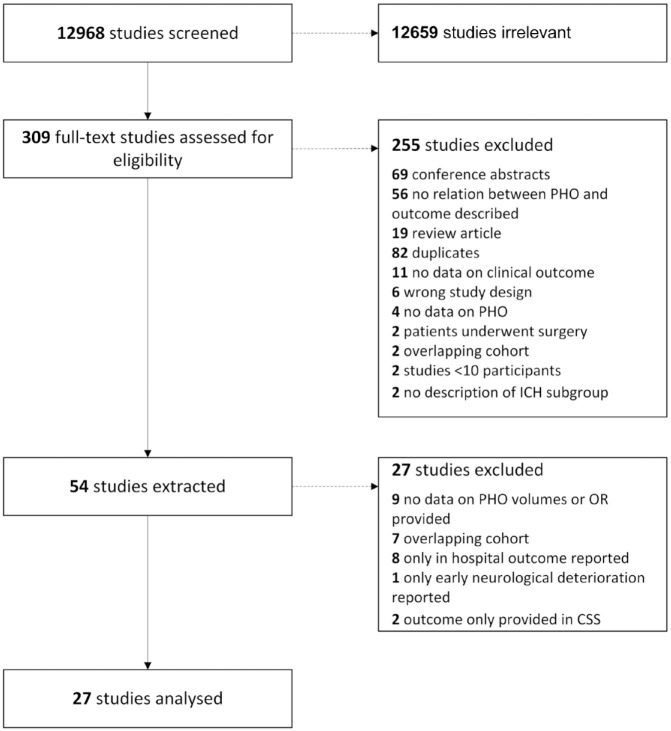
Study selection flow chart. An overview of the 27 excluded studies in the last step is provided in the Supplemental Material.

We identified 12,968 references of which 309 studies were assessed for eligibility. After full text screening of 309 studies, we extracted 54 studies of which we included a total of 27 studies in our analyses with a total of 9534 patients (Figure 1; Supplemental Table 1).^16–42^ Using the NOS scale, 11 studies were classified as of fair quality and 16 studies were deemed of high quality. Median risk of bias score was seven (IQR 6–8; Supplemental Table 2). The most common reason for possible bias was the lack of information on premorbid functional status of the included patients. The main study characteristics are summarised in Table 1.

**Table 1.** Included studies.

Author	Study design	Participants/outcome poor (*N*/*n*)	Age (years)	ICH location (*n*)	ICH volume (mL)	Imaging modality and method of PHO measurement	Measure of PHO	Median PHO volume in poor and good outcome separately	Timing PHO measurement after onset	Definition of (poor) outcome	NOS score
Gebel et al.^19^	Retrospective cohort	48/nr	Mean 62.4 (SD 11.6)	Deep: 36 lobar: 12	Median 12.2 (range 0.4–124.5)	CT, semi-automated computer assisted	aPHO and rPHO	NR	<24 h	mRS 3–6 at 3 months and death at 1 month	6
Alvarez-Sabín et al.^16^	Prospective cohort	21/7	Mean 69.0 (SD 12.92)	Cerebellar, deep, brainstem: nr lobar: 8	Median 18 (IQR 6.3–75.0)	CT, manual segmentation	aPHO	Poor: 3.7 (0–27.7) Good: 6.8 (0.7–17.2)	<24 h	Death at 3 months	5
Delgado et al.^18^	Prospective cohort	78/48	Median 75 (IQR 63–80)	Deep: 58 lobar: 20	Median 17 (IQR 4–38)	CT, manual ABC/2	aPHO	Poor: 10 (1–25) mL Good: 0.94 (0–5) mL	<24 h	mRS 3–6 at 3 months	5
Levine et al.^26^	Case-control	98/nr	NR	Deep: 51 lobar: 47	NR	CT, semi-automated	aPHO	NR	<24 h	Death at 3 months	8
Sansing et al.^35^	Retrospective cohort	287/nr	Mean 66 (SD 12)	Deep/lobar: nr infratentorial: nr	Mean 23.3 (SD 22.8)	96% CT, 4% MRI, semi-automated	aPHO	NR	At 72 h	Worse mRS at 3 months	9
Li et al.^27^	Prospective cohort	59/9	Mean 56 (SD 11)	Deep: 49 lobar: nr infratentorial: nr	Median 10.0 (IQR 5.2–23.9)	85% CT, 15% MRI, manual segmentation	aPHO	Poor: 10.0 (6.7–22.1) Good: 5.7 (2.5–11.7)	<24 h	mRS 4–6 at 3 months	7
Tsai et al.^37^	Prospective cohort	47/29	Mean 65.5 (SD 12.7)	Deep: 40 lobar: 6 cerebellar: 1	Mean 19.6 (SD 13.8)	MRI, manual segmentation	rPHO	Poor: 1.2 (0.8) Good: 1.2 (0.6)	<24 h	mRS 3–6 at 6 months	6
Gupta et al.^20^	Prospective cohort	44/20	Mean 54.95 (SD 9.80)	Deep: 38 lobar: 6	Mean 47.20 (SD 13.07)	CT, semi-automated (Able 3D Doctors)	rPHO	Poor: 0.51 (0.15)^^^ Good: 0.76 (0.24)^^^	24–72 h	mRS 3–6 at 3 months	5
Yang et al.^41^,*	Prospective cohort	1138/627	Mean 65 (SD 13)	Deep: 955 lobar: 120, cerebellar: 31 brainstem: 28	Median 10.4 (IQR 5.4–18.9)	CT, semi-automated threshold-based	aPHO growth	NR	At 24 h	mRS 3–6 at 3 months	8
Murthy et al.^30^	Retrospective cohort	596/367	Median 66.0 (IQR 56.0–75.0)	Deep: 400 lobar: 176 infratentorial: 20	Median 15.0 (IQR 7.9–29.2)	CT, semi-automated planimetry	aPHO, rPHO, growth	NR, growth: Dead: 0.51 mL/h Alive: 0.17 mL/h	<24 h (and at 72 h growth)	mRS 3–6 and death at 3 months	6
Ozdinc et al.^32^	Retrospective cohort	106/43	Median 62 (IQR 44–76)	Deep: 24 lobar: 82	NR	CT, semi-automated	aPHO	Poor: 39.9 (13.3–103.7) Good: 12.1 (11.3–20.1)	<24 h	Death at 30 days	9
Rodriguez-Luna et al.^34^	Retrospective cohort	322/188	Mean 67.8 (SD 15.2)	NR	Median 14.4 (IQR 6.7–28.8)	CT, semi-automated with HU-thresholds	aPHO	Poor: 18.1 (10.3–33.2) Good: 9.5 (5.4–17)	<24 h	mRS 3–6 and death on 3 months	6
Urday et al.^38^	Retrospective cohort	110/91	Mean 71.1 (SD 12.8)	Deep: 59 lobar: 51	Median 19.9 (IQR 8.9–47.9)	CT, manual segmentation	aPHO	NR expansion rates: Poor: 0.22 mL/h Good: 0.02 mL/h	At 72 h	mRS 3–6 at 3 months	7
Wu et al.^40^	Retrospective cohort	861/293	Median 69 (IQR 50–78)	Deep, lobar, cerebellar: nr	Median 14.0 (IQR 6.1–40.1)	CT, semi-automated with HU thresholds	aPHO and OED	NR	72 h	Death at 6 months	9
Iglesias-Rey et al.^24^	Retrospective cohort	887/513	Mean 72.9 (SD 13.1)	Deep: 458 lobar: 328 cerebellar: 46 brainstem: 34	NR	CT, manual ABC/2	aPHO	Poor: 23.1 (25.5)^^^ Good: 8.3 (10.3)^^^	Day 4–7	mRS 3–6 at 3 months	9
Volbers et al.^39^	Retrospective cohort	292/185	Median 70 (IQR 62–78)	Deep: 171 lobar: 121	Median 22.5 (IQR 8.9–46.4)	CT, validated semi-automated	Peak aPHO	Poor: 42.6 (281–67.4) Good: 23.8 (9–45.3)	Day 1–12	mRS 4–6 at 3 months	7
Chen et al.^17^**	Retrospective cohort	131/77	Mean 63 (SD 13)	Deep: 110 lobar: 28	Median 15.6 (IQR 8.0–35.1)	CT, semi-automated (automatic ROI)	aPHO	Poor: 6.1 (1.4–16.3) Good: 2.1 (0.8–4.7)	At 24 h	mRS 3–6 at 3 months	7
Hurford et al.^23^	Retrospective cohort	1028/nr	Mean 64.7 (SD 12.1)	Deep: 869 lobar: 159	Median 15.0 (SD 22.9)	CT, semi-automated planimetry	OED growth	NR	Baseline – 72 h	mRS 3–6 at 3 months	6
Leasure et al.^25^,+	Retrospective cohort	755/286	NR	Deep: 755	NR	CT, semi-automated	aPHO growth	Poor: 1.7 (0.9–3.0) Good: 1.1 (0.6–2.1)	<24 h, at 24 h	mRS 4–6 at 3 months	7
Gusdon et al.^21^	Case-control	80/29	Median 66 (IQR 55.5–73.5)	Deep: 19 lobar: 51 infratentorial: 10	Median 9.1 (IQR 4.68–15.54)	CT, semi-automated	aPHO and growth	NR	<24 h	Death at 1 month	6
Loan et al.^28^	Prospective cohort	342/292	Median 78 (IQR 68–83)	Deep: 138 lobar: 170 infratentorial: 48	Median 22 (IQR 8–51)	CT, semi-automated	aPHO and OED	Poor: 29 (14–55), OED 3.3 (2.6–4.0) Good: 12 (5;18); OED 2.5 (1.8–2.7)	Baseline (<72 h, median 6.5 h)	mRS 3–6 at 1 year	8
Pinho et al.^33^	Retrospective cohort	358/93	Median 71 (IQR 60–80)	Deep: 195 lobar: 114 infratentorial: 50	NR	CT, manual segmentation in ITK SNAP	aPHO and rPHO	NR	<24 h	Death at 1 month	8
Huan et al.^22^	Retrospective cohort	159/77	Median 58.0 (IQR 50.0–66.0)	Deep: 85 lobar: 74	Median 15.4 (IQR 9.6–22.0)	CT, semi-automated plane method	aPHO, rPHO and OED	Poor: 12.0 (8.1–19.2), OED: 9.9 (6.8–10.7). Good: 7.0 (4.2–10.0), OED: 6.1 (4.6–8.1)	72 h	mRS 3–6 at 3 months	6
Lv et al.^29^	Prospective cohort	233/89	Mean 60.2 (range 29–94)	Deep: 200 other: 33	Median 13.4 (IQR 8.8–21.1)	CT, semi-automated computer assisted	aPHO growth	Poor: 7.5 (4.7–14.7) Good: 5.3 (2.6–8.3)	<6 h, at 24 h	mRS 4–6 at 3 months	7
Nawabi et al.^31^	Retrospective cohort	811/586	Median 73 (IQR 60–79)	Deep: 322 lobar: 362 cerebellar: 88 brainstem: 37	Mean 47 (SD 54.11)	CT, semi-automated	aPHO	NR	<24 h	mRS 4–6 at 3 months	8
Shirazian et al.^36^	Retrospective cohort	446/320	Mean 64.9 (SD 15.5)	Deep: 199 lobar: 212 cerebellar: 35	Median 22.5 (IQR 12–40)	CT, ABC/2	aPHO growth	Poor: 44.1 (27.6–70), Good: 19.47 (16–36)	24–48 h	mRS 4–6 at 3 months, death at 1 month	7
Ye et al.^42^	Retrospective cohort	197/99	Mean 59.6 (SD 12.9)	Deep: 145 lobar: 52	Median 12.7 (IQR 5.8–20.9)	CT, semi-automated planimetry	aPHO growth	NR	Baseline – day 3	mRS 4–6 at 3 months	5

ICH: intracerebral haemorrhage; IVH: intraventricular haemorrhage; PHO: perihematomal oedema; NOS: Newcastle Ottawa Scale; NA: not applicable; NR: not reported; SD: standard deviation; IQR: interquartile range; CT: computed tomography scan; MRI: magnetic resonance imaging; mRS: modified Rankin scale score; ROI: region of interest.

* ICH location as reported by the authors (*n* = 1134). This is discrepant with the total study population (*n* = 1138). **ICH location as reported by the authors for the participants included in the outcome analysis (*n* = 131), which differs from the total study population (*n* = 138). ^^^Mean (SD) presented instead of median (IQR). ^+^Leasure et al. is an exploratory analysis of a randomised controlled trial.

Measures of PHO differed between studies (Supplemental Table 3). Twenty-two studies measured aPHO,^16–19,21,22,24,26–40^ with the study mean aPHO ranging from 0.94 to 42.6 mL. Six studies reported rPHO,^19,20,22,30,33,37^ three studies evaluated OED,^22,28,40^ eight studies assessed PHO growth.^21,23,25,29,30,36,41,42^ Ten of the 27 included studies reported on multiple measures of PHO.^19,21,22,28–30,33,36,37,40^ Seventeen studies had an average interval between symptom onset and imaging of 24 h or less,^16–19,21,25–34,37,41^ eight studies performed imaging between 25 and 72 h post onset,^20,22,23,35,36,38,40,42^ one study had an interval longer than 72 h^24^ and one study assessed peak PHO volume between 1 and 12 days after onset.^39^ Twenty-six of 27 studies used CT as their primary imaging modality, the other assessed PHO with MRI.^37^ Outcome measures and follow-up duration were various among studies (Table 1). A total of 20 studies assessed outcome at 3 months after ICH, with poor outcome defined as mRS score 3–6 in 11 of these studies,^17–20,22–24,30,34,38,41^ mRS score 4–6 in seven studies^25,27,29,31,36,39,42^ two reporting death as primary outcome.^16,26^ Six studies assessed clinical outcome at a different intervals, varying between 1 and 12 months post ictus, two of which investigated mRS score 3–6^28,37^ four that assessed death.^21,32,33,40^ Lastly, one study reported clinical outcome only as ‘worse mRS’.^35^

### Primary outcome

**Figure 2.**
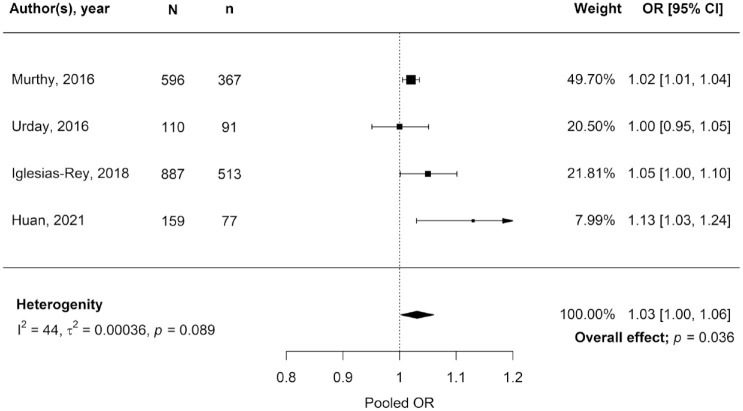
Estimates of the association between absolute perihaematomal oedema volume and poor clinical outcome (mRS score 3–6) at 3 months follow-up. *N:* total participants; *n:* participants with poor outcome; OR: odds ratio; 95% CI: 95% confidence interval. All studies reported an OR adjusted for at least age, ICH volume and clinical condition on admission described as GCS or NIHSS. Murthy et al.^30^ adjusted for an additional five factors, Urday et al.^38^ for two additional factors, Iglesias-Rey et al.^24^ for nine and Huan et al.^22^ for three additional factors (Supplemental Table 4).

In 18 studies (*n* = 7711 patients)^17,18,21–24,27,29–32,34–36,39–42^ a larger volume of PHO (aPHO, rPHO, OED and/or growth) was associated with a higher risk of any type of poor outcome (mRS 3–6, mRS 4–6 and death) at any time, while six studies (*n* = 1633 patients)^16,25,28,33,37,38^ reported a neutral result and three studies (*n* = 190 patients)^19,20,26^ found an association between larger PHO volume and a lower risk of poor outcome. The pooled OR of the four studies reporting the association between PHO measured at any timepoint (all aPHO), and mRS score 3–6 at 3 months was 1.03 (95% CI 1.00–1.06, *p* = 0.036, Figure 2), indicating a 3% increase in the odds of poor functional outcome increases for each mL of aPHO.^38^ In all four studies included in this pooled effect size the OR had been adjusted for multiple factors (Supplemental Table 4), including at least age, ICH volume and severity of ICH by either GCS score or NIHSS score.^22,24,30,38^ Heterogeneity among these four studies was moderate (*I*^2^ 44%). Quality assessment showed an intermediate^22,30^ to low^24,38^ risk of bias in these studies. There was no evidence for publication bias (Supplemental Figure 1).

### Secondary outcomes

Two studies^28,37^ reported on the association between PHO and mRS score 3 and 6 at either 6^37^ or 12 months after ICH.^28^ PHO was measured as either rPHO^37^ or aPHO.^28^ Notably, the study investigating the mRS at 12 months, presented an OR adjusted for ICH volume, age, ICH location, IVH and GCS score,^28^ but the study investigating the mRS at 6 months only provided an unadjusted OR (Supplemental Table 4).^37^ Combining these two studies^28,37^ with the studies included the primary analysis,^22,24,30,38^ we found a pooled OR of 1.02 (95% CI 1.00–1.04, *p* = 0.1, *I*^2^ = 76%) for the association between PHO and mRS 3 and 6 at any time of follow-up.

Seven studies (*n* = 2793 patients)^25,27,29,31,36,39,42^ reported on the influence of PHO and mRS score 4–6 at 3 months. Three of these studies (*n* = 1398)^25,36,42^ assessed PHO growth as their primary analysis, the other four (*n* = 1395)^27,29,31,39^ measured aPHO. One study (*n* = 59 patients)^27^ did not provide an OR but reported that larger PHO on day 3 after admission was associated with mRS 4–6. Of the six studies presenting an OR, four studies presented an OR adjusted for at least ICH volume,^25,36,39,42^ one study^31^ presented an OR that was adjusted for several factors but not for ICH volume and one study^29^ presented an unadjusted OR (Supplemental Table 4). In addition, one of these studies (*n* = 292 patients)^39^ reported that peak aPHO volume was associated with a decreased odds of an mRS 0–3 (aOR 0.984, 95% CI 0.973–0.994, *p* = 0.002) after adjustment for age, ICH volume by location, IVH and NIHSS. Two studies (*n* = 1044 patients)^29,31^ reported an OR (adjusted in one study,^31^Supplemental Table 4), for the association between aPHO and mRS score 4 and 6 after 3 months resulting in a pooled OR of 1.06 (95% CI 0.97–1.17, *p* = 0.21) with considerable heterogeneity (*I*^2^ 93%).

A total of 10 studies (*n* = 2936 patients) reported on the association between PHO volume (aPHO, rPHO, PHO growth or OED) and death.^16,19,21,26,30,32–34,36,40^ Five studies provided only descriptive results.^16,19,32,34,36^ Three of these studies (*n* = 874 patients) reported a significantly higher aPHO in patients that died within one^32,36^ or three^34^ months after ICH while two studies (*n* = 69 patients)^16,19^ reported no statistically significant association between aPHO and death at 3 months. The other five studies provided an adjusted OR and used aPHO as their primary metric with different intervals between ICH and mortality assessment: 1 month in two studies,^21,33^ 3 months in two studies^26,30^ and 6 months in one study.^40^ All five reported ORs were adjusted for multiple factors but at least for ICH volume (Supplemental Table 4). One study reported an OR per 100 cc increase in aPHO, which we have transformed to an OR per mL increase.^26^ We found a pooled OR of 1.02 for death at any time of follow-up (95% CI 0.99–1.05, *p* = 0.14, *I*^2^ = 96%, five studies, Supplemental Figure 2).

**Figure 3.**
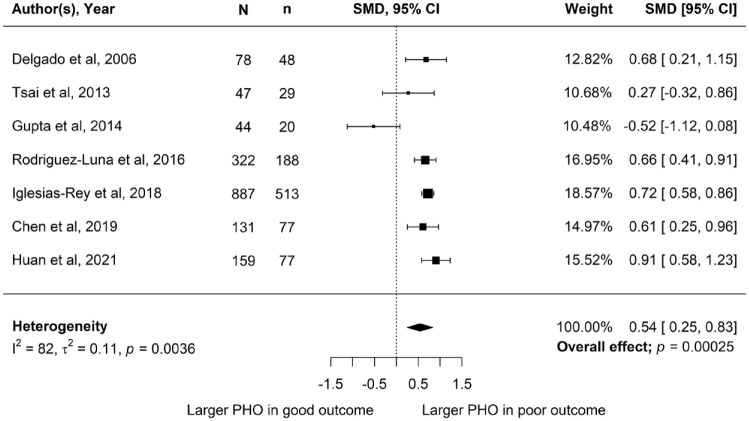
Standardised mean difference in absolute perihaematomal oedema volume between poor clinical outcome (mRS score 3–6) and good outcome (mRS score 0–2) at 3 months follow-up. *N*: total participants; *n*: participants with poor outcome; SMD: standardised mean difference; 95% CI: 95% confidence interval.

Meta-analysis of SMDs in the seven studies (*n* = 1668 patients) reporting aPHO for mRS score 3–6 versus mRS score 0–2 at 3 months revealed a significant difference in mean aPHO, with a higher aPHO in patients with a poor outcome (SMD 0.54, 95% CI 0.25–0.83, *p* = <0.001, *I*^2^ = 82%; Figure 3). Five of the seven studies carried an intermediate risk of bias^18,20,22,34,37^ while the other two were of high quality (Supplemental Table 3).^17,24^

**Figure 4.**
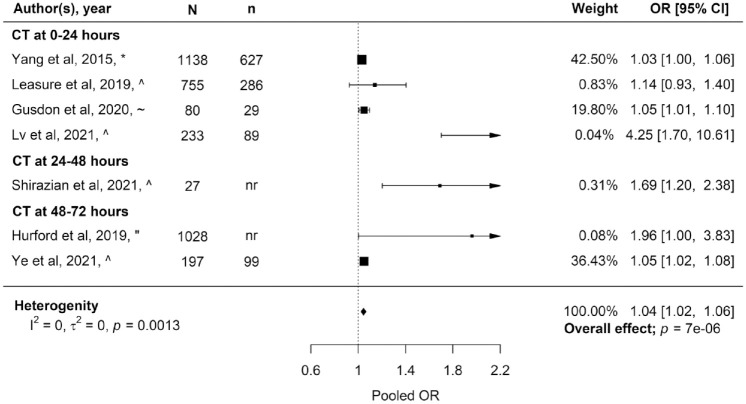
Estimates of the association between perihaematomal oedema growth and any poor outcome at any time of follow-up, stratified by timing of the assessment of growth. *N*: total participants; *n*: participants with poor outcome; OR: odds ratio; 95% CI: 95% confidence interval. *Poor outcome defined as mRS score 3–6 at 3 months after ICH. ^^^Poor outcome defined as mRS score 4–6 at 3 months after ICH. ^~^Poor outcome defined as death at 1 month after ICH. ^"^Poor outcome defined as mRS score 3–6 at 12 months after ICH.

Seven studies (*n* = 4473 patients) assessed the influence of PHO growth on poor functional outcome.^21,23,25,29,36,41,42^ Six out of the seven studies measured aPHO increase while one study assessed increase in OED.^23^ There was large variation in the interval between symptom onset, baseline PHO measurement and the timing of the assessment of PHO growth. Four studies measured PHO growth within the first 24 h,^21,25,29,41^ one study between 24 and 48 h^36^ and two studies between approximately 48 and 72 h after ICH onset.^23,42^ Timing of follow-up and definition of outcome varied between these studies. Studies defined poor outcome as mRS score 3–6^41^ or mRS score 4–6^25,29,36,42^ at 3 months, mRS score 3–6 at 12 months^23^ or as death at 1 month after ICH.^21^ All studies presented ORs adjusted for several factors but at least for ICH volume (Supplemental Table 4). Meta-analysis of the seven studies reporting PHO growth resulted in a pooled OR of 1.04 (95% CI 1.02–1.06, *p* = 0.0013, *I*^2^ = 0%; Figure 4). Sub-analysis of the four studies measuring PHO growth within the first 24 h revealed a pooled OR of 1.04 (95% CI 1.01–1.06, *p* = 0.0019, *I*^2^ = 0.13), while the pooled OR of the three studies measuring PHO growth between 24 and 72 h after symptom onset was 1.40 (95% CI 0.94–2.08, *p* = 0.098, *I*^2^ = 79%).

There was an insufficient number of studies in any of the meta-analyses to perform a meta-regression analysis.

## Discussion

In this systematic review and meta-analysis, we found that in adults with spontaneous ICH, aPHO was associated with poor outcome at 3 months. In addition, PHO growth was associated with poor outcome defined as either mRS 3–6, mRS 4–6 or death.

Secondary brain injury has gained increasing interest as a potential therapeutic target over recent years. However, systematic assessment of the influence of PHO on functional outcome after ICH is hampered by the large variation in methods for PHO measurement and outcome assessment. This heterogeneity also limited a previous systematic review of PHO and outcome, which included 21 articles up to 2016.^12^ In their meta-analysis consisting of just two studies,^30,38^ the authors reported a significant association between aPHO measured at 72 h after ICH onset and poor functional outcome, defined as mRS 3–6, at 90 days (OR 1.02, 95% CI 1.00–1.03, p = 0.007). In comparison to their work, we were able to include multiple new studies and chose to perform a meta-analysis of aPHO at all available timepoints and functional outcome, resulting in increased statistical power.

We found that for every extra mL of aPHO the odds of poor functional outcome after ICH increases with 3%. PHO develops quickly during the first day and increases over the first week after ICH with a reported peak between 7 and 11 days.^8,38^ In our systematic review the majority of studies measured PHO within 24–72 h of ICH onset but there was insufficient data to perform meta-regression to assess the effect of time between symptom onset and PHO measurement on the association between PHO and outcome. Therefore, the effect of peak PHO volume on functional outcome after ICH remains unclear. Future studies of PHO formation could offer valuable insights into the effect of PHO on clinical outcome by measuring PHO at later timepoints as well.

Considering the progressive development of PHO over time, we also assessed the influence of PHO growth on functional outcome. In contrast to the previous meta-analysis,^12^ we found that PHO growth appears to be associated with poor outcome (mRS 3–6, mRS 4–6 and death). This strengthens the hypothesis that PHO formation might be a valuable target to improve outcome after ICH.

Besides the variation in timing of PHO assessment, the studies in this review used a variety of PHO metrics. aPHO volume is traditionally the most frequently used PHO metric. However, it is known that both aPHO and rPHO are strongly correlated with ICH volume. Recently, the OED has been developed as a new PHO metric that is affected less by ICH volume and could reduce the required sample size in clinical trials by as much as 75%.^13^ Only four studies in this review assessed OED in their population, mostly as a secondary measure in addition to aPHO and with different clinical outcome measures. Data was insufficient for separate meta-analysis of OED as PHO metric and functional outcome.

Strengths of our systematic review and meta-analysis include the comprehensive literature search without restrictions in publication language. This resulted in a high number of identified studies and a total number of included patients twice as high as in a previously published systematic review.^12^ Moreover, we applied a generic inverse-variance based random effects model in the meta-analyses, minimising imprecision in the pooled OR estimate. In the analysis of the primary outcome, all ORs that were included were at least adjusted for age, ICH volume and clinical examination by either GCS or NIHSS. In addition, we performed an analysis based on calculated SMDs, in order to consider all the available data even in the absence of ORs. This analysis supported the results of the primary analysis, which strengthens the validity of our findings.

This study also has some limitations. First, quality assessment of the included studies by the NOS revealed a risk of bias almost half of the included studies. Second, meta-analysis was hampered by the variation in applied method and timing of measurements of PHO, and by the variation in the type and timing of outcome measures. Third, not all studies provided ORs and therefore not all studies could be included in the meta-analysis. Calculation of the SMDs partly overcame the small number of ORs reported, but an important limitation of this approach is that for some studies statistical approximation of the mean PHO volume and corresponding SD had to be applied, and we were not able to correct these SMDs for other characteristics that influence the outcome, such as ICH volume. Lastly, due to an insufficient number of studies, we were unable to perform meta-regression to examine potential modifying factors such as age, ICH location or admission blood pressure.

Based on our experience in this systematic review we want to emphasise the importance of uniformity in measurements and reporting of PHO, in the timing of PHO assessment, and in the definition of functional outcome, to allow for optimal comparison of treatment effects. Also, a planned individual patient data meta-analysis of the available literature might provide further insight in the relationship between PHO and functional outcome (PROSPERO CRD42021253263 UK).

Our findings support further research to develop treatment strategies aimed at preventing PHO formation. There is a window of opportunity in the early stage after hospital admission when PHO is developing. Treatments targeting secondary brain injury and PHO formation probably need to be administered during the first days after ICH as PHO continues to develop in this early timeframe. When PHO is assessed as outcome, the timing of its measurement is of great importance. Multiple randomised clinical trials aimed at ameliorating PHO formation are ongoing, investigating IL1-Ra anakinra (NCT04834388), atorvastatin (NCT04857632), fingolimod (NCT04088630) and sodium aescinate (NCT05263167).

## Conclusion

Our data indicates an association between aPHO and PHO growth with poor outcome after ICH. These findings support the development and investigation of new therapeutic interventions targeting PHO formation to evaluate if reduction of PHO leads to improved outcome after ICH. Uniformity in PHO assessment in future studies is of great importance to compare effectiveness of potential new treatment strategies.

## Supplemental Material

sj-docx-1-eso-10.1177_23969873231157884 – Supplemental material for The association between perihaematomal oedema and functional outcome after spontaneous intracerebral haemorrhage: A systematic review and meta-analysis Click here for additional data file. Supplemental material, sj-docx-1-eso-10.1177_23969873231157884 for The association between perihaematomal oedema and functional outcome after spontaneous intracerebral haemorrhage: A systematic review and meta-analysis by Maaike P Cliteur, Lotte Sondag, Laura Cunningham, Rustam Al-Shahi Salman, Neshika Samarasekera, Catharina JM Klijn and Floris HBM Schreuder in European Stroke Journal

sj-docx-2-eso-10.1177_23969873231157884 – Supplemental material for The association between perihaematomal oedema and functional outcome after spontaneous intracerebral haemorrhage: A systematic review and meta-analysis Click here for additional data file. Supplemental material, sj-docx-2-eso-10.1177_23969873231157884 for The association between perihaematomal oedema and functional outcome after spontaneous intracerebral haemorrhage: A systematic review and meta-analysis by Maaike P Cliteur, Lotte Sondag, Laura Cunningham, Rustam Al-Shahi Salman, Neshika Samarasekera, Catharina JM Klijn and Floris HBM Schreuder in European Stroke Journal

## References

[1] Feigin VL Stark BA Johnson CO , et al. Global, regional, and national burden of stroke and its risk factors, 1990-2019: a systematic analysis for the Global Burden of Disease Study 2019. Lancet Neurol 2021; 20: 795–820. 34487721 10.1016/S1474-4422(21)00252-0 PMC8443449

[2] van Asch CJ Luitse MJ Rinkel GJ , et al. Incidence, case fatality, and functional outcome of intracerebral haemorrhage over time, according to age, sex, and ethnic origin: a systematic review and meta-analysis. Lancet Neurol 2010; 9: 167–176. 20056489 10.1016/S1474-4422(09)70340-0

[3] Cordonnier C Demchuk A Ziai W , et al. Intracerebral haemorrhage: current approaches to acute management. Lancet 2018; 392: 1257–1268. 30319113 10.1016/S0140-6736(18)31878-6

[4] Parry-Jones AR Sammut-Powell C Paroutoglou K , et al. An intracerebral hemorrhage care bundle is associated with lower case fatality. Ann Neurol 2019; 86: 495–503. 31291031 10.1002/ana.25546 PMC6771716

[5] Wilkinson DA Pandey AS Thompson BG , et al. Injury mechanisms in acute intracerebral hemorrhage. Neuropharmacology 2018; 134: 240–248. 28947377 10.1016/j.neuropharm.2017.09.033 PMC6027647

[6] Askenase MH Sansing LH . Stages of the inflammatory response in pathology and tissue repair after intracerebral hemorrhage. Semin Neurol 2016; 36: 288–297. 27214704 10.1055/s-0036-1582132 PMC4956485

[7] Appelboom G Bruce SS Hickman ZL , et al. Volume-dependent effect of perihaematomal oedema on outcome for spontaneous intracerebral haemorrhages. J Neurol Neurosurg Psychiatry 2013; 84: 488–493. 23345281 10.1136/jnnp-2012-303160

[8] Chen Y Chen S Chang J , et al. Perihematomal edema after intracerebral hemorrhage: an update on pathogenesis, risk factors, and therapeutic advances. Front Immunol 2021; 12: 740632. 34737745 10.3389/fimmu.2021.740632 PMC8560684

[9] Jeon H Kim M Park W , et al. Upregulation of AQP4 improves blood-brain barrier integrity and perihematomal edema following intracerebral hemorrhage. Neurotherapeutics 2021; 18: 2692–2706. 34545550 10.1007/s13311-021-01126-2 PMC8804112

[10] Lee SH Park HK Ryu WS , et al. Effects of celecoxib on hematoma and edema volumes in primary intracerebral hemorrhage: a multicenter randomized controlled trial. Eur J Neurol 2013; 20: 1161–1169. 23551657 10.1111/ene.12140

[11] Selim M Norton C . Perihematomal edema: implications for intracerebral hemorrhage research and therapeutic advances. J Neurosci Res 2020; 98: 212–218. 30575082 10.1002/jnr.24372 PMC6588515

[12] Yu Z Ma L Zheng J , et al. Prognostic role of perihematomal edema in intracerebral hemorrhage: a systematic review. Turk Neurosurg. Epub ahead of print 25 January 2017. DOI: 10.5137/1019-5149.JTN.19659-16.0. 28266005

[13] Parry-Jones AR Wang X Sato S , et al. Edema extension distance: outcome measure for phase II clinical trials targeting edema after intracerebral hemorrhage. Stroke 2015; 46: e137–e140. 10.1161/STROKEAHA.115.008818 25944323

[14] Shah VA Thompson RE Yenokyan G , et al. One-year outcome trajectories and factors associated with functional recovery among survivors of intracerebral and intraventricular hemorrhage with initial severe disability. JAMA Neurol 2022; 79: 856–868. 35877105 10.1001/jamaneurol.2022.1991 PMC9316056

[15] Wan X Wang W Liu J , et al. Estimating the sample mean and standard deviation from the sample size, median, range and/or interquartile range. BMC Med Res Methodol 2014; 14: 135. 25524443 10.1186/1471-2288-14-135 PMC4383202

[16] Alvarez-Sabín J Delgado P Abilleira S , et al. Temporal profile of matrix metalloproteinases and their inhibitors after spontaneous intracerebral hemorrhage: relationship to clinical and radiological outcome. Stroke 2004; 35: 1316–1322. 15087562 10.1161/01.STR.0000126827.69286.90

[17] Chen L Xu M Yan S , et al. Insufficient cerebral venous drainage predicts early edema in acute intracerebral hemorrhage. Neurology 2019; 93: e1463–e1473. 10.1212/WNL.0000000000008242 31492719

[18] Delgado P Alvarez Sabin J Santamarina E , et al. Plasma S100B level after acute spontaneous intracerebral hemorrhage. Stroke 2006; 37: 2837–2839. 17008613 10.1161/01.STR.0000245085.58807.ad

[19] Gebel JM Jr Jauch EC Brott TG , et al. Relative edema volume is a predictor of outcome in patients with hyperacute spontaneous intracerebral hemorrhage. Stroke 2002; 33: 2636–2641. 12411654 10.1161/01.str.0000035283.34109.ea

[20] Gupta M Verma R Parihar A , et al. Perihematomal edema as predictor of outcome in spontaneous intracerebral hemorrhage. J Neurosci Rural Pract 2014; 5: 48–54. 24741251 10.4103/0976-3147.127873 PMC3985358

[21] Gusdon AM Nyquist PA Torres-Lopez VM , et al. Perihematomal edema after intracerebral hemorrhage in patients with active malignancy. Stroke 2020; 51: 129–136. 31744426 10.1161/STROKEAHA.119.027085 PMC7048624

[22] Huan R Li Y Tan J , et al. The Hounsfield unit of perihematomal edema is associated with poor clinical outcomes in intracerebral hemorrhage. World Neurosurg 2021; 146: e829–e836. 10.1016/j.wneu.2020.11.025 33189917

[23] Hurford R Vail A Heal C , et al. Oedema extension distance in intracerebral haemorrhage: association with baseline characteristics and long-term outcome. Eur Stroke J 2019; 4: 263–270. 31984234 10.1177/2396987319848203 PMC6960688

[24] Iglesias-Rey R Rodríguez-Yáñez M Arias S , et al. Inflammation, edema and poor outcome are associated with hyperthermia in hypertensive intracerebral hemorrhages. Eur J Neurol 2018; 25: 1161–1168. 29751370 10.1111/ene.13677 PMC6099376

[25] Leasure AC Qureshi AI Murthy SB , et al. Intensive blood pressure reduction and perihematomal edema expansion in deep intracerebral hemorrhage. Stroke 2019; 50: 2016–2022. 31272326 10.1161/STROKEAHA.119.024838 PMC6646091

[26] Levine JM Snider R Finkelstein D , et al. Early edema in warfarin-related intracerebral hemorrhage. Neurocrit Care 2007; 7: 58–63. 17657657 10.1007/s12028-007-0039-3

[27] Li N Liu YF Ma L , et al. Association of molecular markers with perihematomal edema and clinical outcome in intracerebral hemorrhage. Stroke 2013; 44: 658–663. 23391772 10.1161/STROKEAHA.112.673590

[28] Loan JJ Gane AB Middleton L , et al. Association of baseline hematoma and edema volumes with one-year outcome and long-term survival after spontaneous intracerebral hemorrhage: a community-based inception cohort study. Int J Stroke 2021; 16: 828–839. 34165016 10.1177/1747493020974282 PMC8521378

[29] Lv XN Li ZQ Deng L , et al. Early perihematomal edema expansion: definition, significance, and association with outcomes after intracerebral hemorrhage. Oxid Med Cell Longev 2021; 2021: 6249509. 34552686 10.1155/2021/6249509 PMC8452407

[30] Murthy SB Urday S Beslow LA , et al. Rate of perihaematomal oedema expansion is associated with poor clinical outcomes in intracerebral haemorrhage. J Neurol Neurosurg Psychiatry 2016; 87: 1169–1173. 27466360 10.1136/jnnp-2016-313653 PMC5299159

[31] Nawabi J Elsayed S Morotti A , et al. Perihematomal edema and clinical outcome in intracerebral hemorrhage related to different oral anticoagulants. J Clin Med 2021; 10: 2234. 34063991 10.3390/jcm10112234 PMC8196746

[32] Ozdinc S Unlu E Karakaya Z , et al. Prognostic value of perihematomal edema area at the initial ED presentation in patients with intracranial hematoma. Am J Emerg Med 2016; 34: 1241–1246. 27085454 10.1016/j.ajem.2016.03.048

[33] Pinho J Silva L Quintas-Neves M , et al. Red cell distribution width is associated with 30-day mortality in patients with spontaneous intracerebral hemorrhage. Neurocrit Care 2021; 34: 825–832. 32959199 10.1007/s12028-020-01103-1 PMC8179905

[34] Rodriguez-Luna D Stewart T Dowlatshahi D , et al. Perihematomal edema is greater in the presence of a spot sign but does not predict intracerebral hematoma expansion. Stroke 2016; 47: 350–355. 26696644 10.1161/STROKEAHA.115.011295

[35] Sansing LH Messe SR Cucchiara BL , et al. Anti-adrenergic medications and edema development after intracerebral hemorrhage. Neurocrit Care 2011; 14: 395–400. 21264527 10.1007/s12028-010-9498-z

[36] Shirazian A Peralta-Cuervo AF Aguilera-Pena MP , et al. Sustained low-efficiency dialysis is associated with worsening cerebral edema and outcomes in intracerebral hemorrhage. Neurocrit Care 2021; 35: 221–231. 33403579 10.1007/s12028-020-01155-3

[37] Tsai YH Hsu LM Weng HH , et al. Functional diffusion map as an imaging predictor of functional outcome in patients with primary intracerebral haemorrhage. Br J Radiol 2013; 86: 20110644. 23255534 10.1259/bjr.20110644 PMC4651067

[38] Urday S Beslow LA Dai F , et al. Rate of perihematomal edema expansion predicts outcome after intracerebral hemorrhage. Crit Care Med 2016; 44: 790–797. 26757167 10.1097/CCM.0000000000001553 PMC4859217

[39] Volbers B Giede-Jeppe A Gerner ST , et al. Peak perihemorrhagic edema correlates with functional outcome in intracerebral hemorrhage. Neurology 2018; 90: e1005–e1012. 10.1212/WNL.0000000000005167 29453243

[40] Wu TY Sharma G Strbian D , et al. Natural history of perihematomal edema and impact on outcome after intracerebral hemorrhage. Stroke 2017; 48: 873–879. 28275199 10.1161/STROKEAHA.116.014416

[41] Yang J Arima H Wu G , et al. Prognostic significance of perihematomal edema in acute intracerebral hemorrhage: pooled analysis from the intensive blood pressure reduction in acute cerebral hemorrhage trial studies. Stroke 2015; 46: 1009–1013. 25712944 10.1161/STROKEAHA.114.007154

[42] Ye G Huang S Chen R , et al. Early predictors of the increase in perihematomal edema volume after intracerebral hemorrhage: a retrospective analysis from the Risa-MIS-ICH study. Front Neurol 2021; 12: 700166. 34385972 10.3389/fneur.2021.700166 PMC8353085

