# Supplementary material for: The association between perihaematomal oedema and functional outcome after spontaneous intracerebral haemorrhage: A systematic review and meta-analysis
Source: Eur Stroke J. 2023 Feb 18;8(2):423–33. doi: 10.1177/23969873231157884 (PMC10334181; doi:10.1177/23969873231157884)
Supplement: sj-docx-2-eso-10.1177_23969873231157884 – Supplemental material for The association between perihaematomal oedema and functional outcome after spontaneous intracerebral haemorrhage: A systematic review and meta-analysis [file sj-docx-2-eso-10.1177_23969873231157884.docx]

**SUPPLEMENTARY DATA to ‘The association between perihaematomal oedema and functional outcome after spontaneous intracerebral haemorrhage: a systematic review and meta-analysis.’**

**Search strategy**

1. brain edema/
2. (brain ?edema or perih?ematomal ?edema or perih?emorrhagic ?edema or cytotoxic ?edema or perih?ematomal hypodensity or perih?ematomal brain ?edema or perih?ematomal ?edema absolute volume or PHE absolute volume or perih?ematomal ?edema expansion rate or PHEER or perih?ematomal ?edematous region).ab,ti.
3. 1 or 2
4. ((brain$ or cerebr$ or cerebell$ or intracerebr$ or intracran$ or parenchyma$ or intraparenchyma$ or intraventricular or infratentorial or supratentorial or basal gang$ or ganglion$ or putaminal or putamen or posterior fossa or hemispher$ or stroke or apoplex$ or brain?stem or intra?axial or lobar or deep or thalam$ or cortical or superficial or vertebrobasil$ or front$ or tempor$ or pariet$ or occipit$) adj5 (h?emorrhag$ or h?ematoma$ or bleed$)).ab,ti.
5. (h?emorrhag$ adj (stroke$ or cerebrovasc$ or cerebr?vasc$ or cerebral vasc$ or brain vasc$ or cva$ or apoplex$ or attack$ or event$ or insult$)).ab,ti.
6. exp basal ganglion hemorrhage/ or exp cerebral hemorrhage/ or exp intracranial hemorrhages/ or intracranial haemorrhage, hypertensive/
7. 4 or 5 or 6
8. 3 and 7
9. 8 not (Comment/ or Editorial/ or Letter/)
10. 9 not ((exp animal/ or nonhuman/) not exp human/)

**Supplementary Table 1: Characteristics of the 27 studies that were excluded after full-text extraction**

| **Author (year)** | **Study design** | **Participants/ poor outcome (N/n)** | **Measure of PHO** | **Definition of poor outcome** | **Effect found** | **Reason for exclusion** |
| --- | --- | --- | --- | --- | --- | --- |
| Al Khaled 2014^1^ | Prospective cohort | 309/55 | Presence of PHO | Death at 3 months (in hospital survivors) | 3-month mortality correlated significantly with presence of oedema (OR 5.7 (2.3-13.8)) | Only presence or absence of PHO reported |
| Leira 2004^2^ | Prospective cohort | 266/61 | Perihaematomal hypodensity | Early neurological deterioration CSS increase at 48 hours | Significantly larger hypodensity volume in patients with early deterioration (p=0.007) | No OR or PHO volumes reported. Only association with in hospital early neurological deterioration on Canadian stroke scale reported |
| Li 2013 (2)^3^ | Prospective cohort | 21/9 | aPHO at 3 days | mRS at 90 days | Larger PHO at 3 days was associated with poor outcome (p=0.07) | No PHO volumes or OR reported, only presence or absence of PHO growth |
| Puig 2019^4^ | Prospective cohort | 43/19 | aPHO | mRS 3-6 at 3 months | No significant difference in aPHO at admission or at 24 hours | No PHO volumes or OR reported |
| Roy-O’Reilly 2017^5^ | Prospective cohort | 47/NR | PHO expansion | mRS at discharge and 90 days | Patients with high PHO expansion (>10cm3 increase) had poorer mRS at discharge (p=0.024) | Only peak PHO reported at unclear time window |
| Sansing 2003^6^ | Retrospective cohort | 45/29 | PHO growth | mRS 3-6 at discharge | OR 1.03 (1.00-1.07) for oedema vs discharge disposition | Only PHO growth presented |
| Kumar 2016^7^ | Prospective cohort | 158/NR aimed (actual NR) | aPHO | Death or GCS 3-5 at 24 hours | Significantly larger aPHO volume in deaths compared to alive | No PHO volumes or OR available, unknown timing of outcome assessment |
| Venkatasubramanian 2011^8^ | Prospective cohort | 27/NR | PHO growth first MRI to peak | NR, median mRS at 3 months | PHO growth correlated with decline neurological status at 48 hours but not with 3 months functional outcome | No PHO volumes or OR available for good and poor outcome groups |
| Zubkov 2008^9^ | Retrospective cohort | 83/46 | PHO growth | mRS 4-6 at 1 year | No significant difference in PHO expansion (all warfarin-related ICH) | No PHO volumes and no OR reported |
|  |  |  |  |  |  |  |
| Arima 2009^10^ | Patients from RCT | 270/119 | a/rPHO increase  (bl, 24hrs and 72hrs) | mRS 3-6 at 90 days | Absolute and relative increases in PHO growth were significantly associated with poor outcome, but not after adjustment for baseline ICH volume | Overlapping cohort/dataset with Yang |
| Grunwald 2017^11^ | Retrospective cohort | 115/59 | a/rPHO growth | Death, mRS 3-6 at 3 months | PHO expansion at 24 hours was associated with mortality for deep (p=0.03) and lobar (p=0.02) ICH; PHO expansion in deep ICH at 72 hours was associated with poor outcome (OR 4.04 (1.25-13.04)), not for lobar ICH. | Overlapping cohort/dataset with Urday but in Grunwald deep vs lobar and using expansion rate |
| Hervella 2020^12^ | Retrospective cohort (from prospective cohort) | 795/447 | Perihaema-tomal hypodensity | mRS 3-6 at discharge and at 3 months | Patients with poor outcome had significantly larger perihaematomal hypodensity volumes | Overlapping cohort/dataset with Iglesias |
| Lord 2015^13^ | Retrospective cohort from prospective database | 376/176 | aPHO baseline and 72hrs | Neurological deterioration (GCS or NIHSS) <15 days | Subacute neurological deterioration was associated with PHO at 72hrs (OR 1.03/mL (1.02-1.05) | Overlapping cohort (VISTA database) with Murthy and Lord reported only early neurological deterioration |
| Sprugel 2019^14^ | Retrospective from prospective cohort | 300/NR | Peak a/rPHO | mRS 4-6 at 3 months | Peak PHO was associated with worse functional outcome in ICH <30mL (OR 2.63 (1.68-4.21)) but not in ICH>30mL (OR 1.20 (0.88-1.63)) | Overlapping cohort/dataset with Volbers 2018 |
| Staykov 2011^15^ | Retrospective cohort | 219/14 | Peak aPHO | In hospital mortality | Increase in aPHO from day 1 to 3 was significantly predictive for in hospital mortality (Exp B 1.04, p=0.014) | Overlapping cohort/dataset with Volbers 2018 |
| Volbers 2016^16^ | Retrospective cohort | 310/200 | (peak) aPHO | mRS 4-6 at discharge | High peak PHO predicted poor outcome at discharge (OR 0.977 (0.957-0.998) | Overlapping cohort/dataset with Volbers 2018 |
|  |  |  |  |  |  |  |
| Appelboom 2013^17^ | Prospective cohort | 133/100 | a/rPHO | mRS 4-6 at discharge | No significant association between rPHO and outcome, strong association between aPHO and outcome (p=0.009), adjusted in patients <30cm3: OR1.123 (1.021-1.273) | Only in hospital mRS reported |
| Bakshayesh 2014^18^ | Prospective cohort | 98/30 | aPHO | In hospital mortality | After multivariable analysis PHO was an independent predictor of mortality (OR 0.75 (0.60-0.93) | Only in hospital mortality reported |
| Inaji 2003^19^ | Prospective cohort | 14/NR | PHO day 1,3,7,14,28 | In hospital NIHSS change | No relationship between PHO and outcome was reported | Only in hospital NIHSS change |
| Fainardi 2013^20^ | Prospective cohort | 35/NR | aPHO, PHO growth | NIHSS increase >3 points at 7 days | More frequent neurological worsening in patients with than in patients without perihaematomal vasogenic rADC values on MRI at day 7 (p<0.02) | Only 7 days secondary subacute neurological worsening as outcome reported |
| McCarron 1999^21^ | Prospective cohort | 102/29 | aPHO | In hospital mortality | Significantly larger aPHO volume in poor outcome group (p<0.01) | Only in hospital death reported as outcome |
| Peng 2019^22^ | Retrospective cohort | 121/NR | Delayed aPHO | mRS 2-6 at discharge | Delayed PHO was a risk factor for poor prognosis (OR 3.06 (1.20-7.84) | Only outcome (mRS) at discharge available |
| Suarez-Pinilla 2014^23^ | Retrospective cohort | 63/43 | aPHO | Death, mRS 4-6 at discharge | in hospital mortality related to PHO in VKA-related ICH (p=0.036) | Only in hospital death and mRS reported |
| Stosser 2016^24^ | Retrospective cohort | 169/40 | a/r peri-haematomal diffusion restriction | Death (mRS 4-6) at discharge | Occurrence of perihaematomal diffusion restriction was associated with poor outcome, but no independent predictor. Significantly higher PHO volume in deaths (p<0.001) | Only in hospital death and mRS reported |
|  |  |  |  |  |  |  |
| Sykora 2009^25^ | Prospective cohort from prospective registry | 38/8 | rPHO | Early neurological deterioration, increase NIHSS >3 points | Univariate: oedema (p=0.006) correlated with deterioration, after logistic regression remained significant (OR 22.6 (2.2-232.5) | Only early neurological deterioration reported as outcome |
|  |  |  |  |  |  |  |
| Castillo 2002^26^ | Prospective cohort | 124/53 | Perihaematomal hypodensity | CSS<7 at 3 months | No significant difference between volume hypodensity at day 3-4, significant higher at 3 months in poor outcome group (p<0.001) | Canadian stroke scale as outcome measure |
| Rendevski 2018^27^ | Prospective cohort | 50/23 | aPHO | CSS<7 ar 3 months | Significantly higher aPHO after 5 days volume in poor outcome (p0.005) | Canadian stroke scale as outcome measure |

Studies are categorised by reason of exclusion. Green studies found a positive correlation between PHO and outcome, or a significantly higher PHO volume in the poor outcome group. Blue studies found no correlation or significant difference or did not report on this. Red studies found an inverse association (or larger PHO volumes in the good outcome groups). Abbreviations: GCS, Glasgow Coma Scale score; ICH, intracerebral haemorrhage; mRS, modified Rankin Scale score; NIHSS, National Institutes of Healthcare Stroke Scale score; OED, oedema extension distance; OR, odds ratio; PHO, perihaematomal oedema (aPHO, absolute PHO and rPHO relative PHO).

**References**

1. Al-Khaled M, Eggers J and Qug SSS. Prognosis of intracerebral hemorrhage after conservative treatment. *J Stroke Cerebrovasc Dis* 2014; 23: 230-234. 20130124. DOI: 10.1016/j.jstrokecerebrovasdis.2012.12.018.

2. Leira R, Davalos A, Silva Y, et al. Early neurologic deterioration in intracerebral hemorrhage: predictors and associated factors. *Neurology* 2004; 63: 461-467. DOI: 10.1212/01.wnl.0000133204.81153.ac.

3. Li N, Worthmann H, Heeren M, et al. Temporal pattern of cytotoxic edema in the perihematomal region after intracerebral hemorrhage: a serial magnetic resonance imaging study. *Stroke* 2013; 44: 1144-1146. 20130207. DOI: 10.1161/STROKEAHA.111.000056.

4. Puig J, Blasco G, Terceno M, et al. Predicting Motor Outcome in Acute Intracerebral Hemorrhage. *AJNR Am J Neuroradiol* 2019; 40: 769-775. 20190418. DOI: 10.3174/ajnr.A6038.

5. Roy-O'Reilly M, Zhu L, Atadja L, et al. Soluble CD163 in intracerebral hemorrhage: biomarker for perihematomal edema. *Ann Clin Transl Neurol* 2017; 4: 793-800. 20171019. DOI: 10.1002/acn3.485.

6. Sansing LH, Kaznatcheeva EA, Perkins CJ, et al. Edema after intracerebral hemorrhage: correlations with coagulation parameters and treatment. *J Neurosurg* 2003; 98: 985-992. DOI: 10.3171/jns.2003.98.5.0985.

7. Sai Sampath Kumar N SG, Anil Kumar Thatikonda, Ravi kiran Padala, T Sunanda. Predictors of Mortality of Primary Intracerebral Hemorrhage among the Sea Coast

Population of South India. *Mathews J Neurol* 2016; 1: 005.

8. Venkatasubramanian C, Mlynash M, Finley-Caulfield A, et al. Natural history of perihematomal edema after intracerebral hemorrhage measured by serial magnetic resonance imaging. *Stroke* 2011; 42: 73-80. 20101216. DOI: 10.1161/STROKEAHA.110.590646.

9. Zubkov AY, Mandrekar JN, Claassen DO, et al. Predictors of outcome in warfarin-related intracerebral hemorrhage. *Arch Neurol* 2008; 65: 1320-1325. DOI: 10.1001/archneur.65.10.1320.

10. Arima H, Wang JG, Huang Y, et al. Significance of perihematomal edema in acute intracerebral hemorrhage: the INTERACT trial. *Neurology* 2009; 73: 1963-1968. DOI: 10.1212/WNL.0b013e3181c55ed3.

11. Grunwald Z, Beslow LA, Urday S, et al. Perihematomal Edema Expansion Rates and Patient Outcomes in Deep and Lobar Intracerebral Hemorrhage. *Neurocrit Care* 2017; 26: 205-212. DOI: 10.1007/s12028-016-0321-3.

12. Hervella P, Rodriguez-Yanez M, Pumar JM, et al. Antihyperthermic treatment decreases perihematomal hypodensity. *Neurology* 2020; 94: e1738-e1748. 20200327. DOI: 10.1212/WNL.0000000000009288.

13. Lord AS, Gilmore E, Choi HA, et al. Time course and predictors of neurological deterioration after intracerebral hemorrhage. *Stroke* 2015; 46: 647-652. 20150205. DOI: 10.1161/STROKEAHA.114.007704.

14. Sprugel MI, Kuramatsu JB, Volbers B, et al. Perihemorrhagic edema: Revisiting hematoma volume, location, and surface. *Neurology* 2019; 93: e1159-e1170. 20190816. DOI: 10.1212/WNL.0000000000008129.

15. Staykov D, Wagner I, Volbers B, et al. Natural course of perihemorrhagic edema after intracerebral hemorrhage. *Stroke* 2011; 42: 2625-2629. 20110707. DOI: 10.1161/STROKEAHA.111.618611.

16. Volbers B, Willfarth W, Kuramatsu JB, et al. Impact of Perihemorrhagic Edema on Short-Term Outcome After Intracerebral Hemorrhage. *Neurocrit Care* 2016; 24: 404-412. DOI: 10.1007/s12028-015-0185-y.

17. Appelboom G, Bruce SS, Hickman ZL, et al. Volume-dependent effect of perihaematomal oedema on outcome for spontaneous intracerebral haemorrhages. *J Neurol Neurosurg Psychiatry* 2013; 84: 488-493. 20130123. DOI: 10.1136/jnnp-2012-303160.

18. Bakhshayesh B, Hosseininezhad M, Seyed Saadat SM, et al. Predicting in-hospital mortality in Iranian patients with spontaneous intracerebral hemorrhage. *Iran J Neurol* 2014; 13: 231-236.

19. Inaji M, Tomita H, Tone O, et al. Chronological changes of perihematomal edema of human intracerebral hematoma. *Acta Neurochir Suppl* 2003; 86: 445-448. DOI: 10.1007/978-3-7091-0651-8_91.

20. Fainardi E, Borrelli M, Saletti A, et al. Temporal changes in perihematomal apparent diffusion coefficient values during the transition from acute to subacute phases in patients with spontaneous intracerebral hemorrhage. *Neuroradiology* 2013; 55: 145-156. 20120918. DOI: 10.1007/s00234-012-1093-x.

21. McCarron MO, Hoffmann KL, DeLong DM, et al. Intracerebral hemorrhage outcome: apolipoprotein E genotype, hematoma, and edema volumes. *Neurology* 1999; 53: 2176-2179. DOI: 10.1212/wnl.53.9.2176.

22. Peng WJ, Li Q, Tang JH, et al. The risk factors and prognosis of delayed perihematomal edema in patients with spontaneous intracerebral hemorrhage. *CNS Neurosci Ther* 2019; 25: 1189-1194. 20190922. DOI: 10.1111/cns.13219.

23. Suarez-Pinilla M, Fernandez-Rodriguez A, Benavente-Fernandez L, et al. Vitamin K antagonist-associated intracerebral hemorrhage: lessons from a devastating disease in the dawn of the new oral anticoagulants. *J Stroke Cerebrovasc Dis* 2014; 23: 732-742. 20130815. DOI: 10.1016/j.jstrokecerebrovasdis.2013.06.034.

24. Stosser S, Neugebauer H, Althaus K, et al. Perihematomal Diffusion Restriction in Intracerebral Hemorrhage Depends on Hematoma Volume, But Does Not Predict Outcome. *Cerebrovasc Dis* 2016; 42: 280-287. 20160525. DOI: 10.1159/000446549.

25. Sykora M, Diedler J, Turcani P, et al. Subacute perihematomal edema in intracerebral hemorrhage is associated with impaired blood pressure regulation. *J Neurol Sci* 2009; 284: 108-112. 20090509. DOI: 10.1016/j.jns.2009.04.028.

26. Castillo J, Davalos A, Alvarez-Sabin J, et al. Molecular signatures of brain injury after intracerebral hemorrhage. *Neurology* 2002; 58: 624-629. DOI: 10.1212/wnl.58.4.624.

27. Rendevski V, Aleksovski B, Stojanov D, et al. Modeling prognostic factors for poor neurological outcome in conservatively treated patients with intracerebral hemorrhage: A focus on TNF-alpha. *Clin Neurol Neurosurg* 2018; 172: 51-58. 20180628. DOI: 10.1016/j.clineuro.2018.06.027.

**Supplementary Table 2: Quality assessment of the studies included in the systematic review and meta-analysis**

| **a. Risk of bias assessment in cohort studies (Newcastle-Ottawa Quality Assessment Scale)** | | | | | | | | | | | | | | | | | | |  | |
| --- | --- | --- | --- | --- | --- | --- | --- | --- | --- | --- | --- | --- | --- | --- | --- | --- | --- | --- | --- | --- |
| **Author** | | **Year** | **Representativeness exposed cohort** | | | **Selection non-exposed cohort** | | **Ascertainment of exposure** | | **Outcome not present at start** | | **Comparability** | | **Outcome assessment** | | **Adequacy follow-up** | | **Follow-up long enough for outcome to occur** | **Total score** |  |
| Gebel | | 2002 | High | | | Low | | Low | | High | | Low** | | High | | Low | | Low | 6 |  |
| Alvarez-Sabin | | 2004 | Low | | | Low | | Low | | High | | High | | High | | Low | | Low | 5 |  |
| Delgado | | 2006 | Low | | | Low | | Low | | High | | High | | High | | Low | | Low | 5 |  |
| Sansing | | 2011 | Low | | | Low | | Low | | Low | | Low** | | Low | | Low | | Low | 9 |  |
| Li | | 2013 | Low | | | Low | | Low | | High | | Low** | | High | | Low | | Low | 7 |  |
| Tsai | | 2013 | High | | | Low | | Low | | High | | Low* | | Low | | Low | | Low | 6 |  |
| Gupta | | 2014 | High | | | Low | | Low | | High | | Low* | | High | | Low | | Low | 5 |  |
| Yang | | 2015 | Low | | | Low | | Low | | High | | Low** | | Low | | Low | | Low | 8 |  |
| Murthy | | 2016 | High | | | Low | | Low | | High | | Low** | | High | | Low | | Low | 6 |  |
| Ozdinic | | 2016 | Low | | | Low | | Low | | Low | | Low** | | Low | | Low | | Low | 9 |  |
| Rodriguez-Luna | | 2016 | Low | | | Low | | Low | | Low | | High | | High | | Low | | Low | 6 |  |
| Urday | | 2016 | Low | | | Low | | Low | | High | | Low** | | Low | | High | | Low | 7 |  |
| Wu | | 2017 | Low | | | Low | | Low | | Low | | Low** | | Low | | Low | | Low | 9 |  |
| Iglesias-Rey | | 2018 | Low | | | Low | | Low | | Low | | Low** | | Low | | Low | | Low | 9 |  |
| Volbers | | 2018 | Low | | | Low | | Low | | Low | | Low* | | High | | Low | | Low | 7 |  |
| Chen | | 2019 | High | | | Low | | Low | | High | | Low** | | Low | | Low | | Low | 7 |  |
| Hurford | | 2019 | High | | | Low | | Low | | Low | | Low* | | High | | Low | | Low | 6 |  |
| Leasure | | 2019 | High | | | Low | | Low | | High | | Low** | | Low | | Low | | Low | 7 |  |
| Pinho | | 2020 | High | | | Low | | Low | | Low | | Low** | | Low | | Low | | Low | 8 |  |
| Huan | | 2021 | High | | | Low | | Low | | High | | Low* | | Low | | Low | | Low | 6 |  |
| Loan | | 2021 | Low | | | Low | | Low | | High | | Low** | | Low | | Low | | Low | 8 |  |
| Lv | | 2021 | High | | | Low | | Low | | High | | Low** | | Low | | Low | | Low | 7 |  |
| Nawabi | | 2021 | Low | | | Low | | Low | | High | | Low** | | Low | | Low | | Low | 8 |  |
| Shirazian | | 2021 | Low | | | Low | | Low | | Low | | High | | Low | | Low | | Low | 7 |  |
| Ye | | 2021 | High | | | Low | | Low | | High | | Low* | | Low | | High | | Low | 5 |  |
| **b. Risk of bias assessment in case-control studies (Newcastle-Ottawa Quality Assessment Scale)** | | | | | | | | | | | | | | | | | | | | |
| **Author** | **Year** | | | **Selection of controls** | **Ascertainment of exposure** | | **Adequate case definition** | | **Representativeness cases** | | **Definition controls** | | **Comparability** | | **Non-response rate** | | **Same method ascertainment controls and cases** | | **Total score** | |
| Levine | 2007 | | | Low | Low | | Low | | Low | | Low | | Low* | | Low | | Low | | 8 | |
| Gusdon | 2020 | | | High | Low | | Low | | High | | Low | | Low* | | High | | Low | | 6 | |

***** One point rewarded for comparability, with a maximum score of two on this item, ** two points rewarded for comparability, with a maximum score of two on this item.

**Supplementary Figure 1: Funnel plot regarding publication bias in the four studies reporting an odds ratio on aPHO measured at any timepoint, and mRS score 3-6 at 3 months.**

**
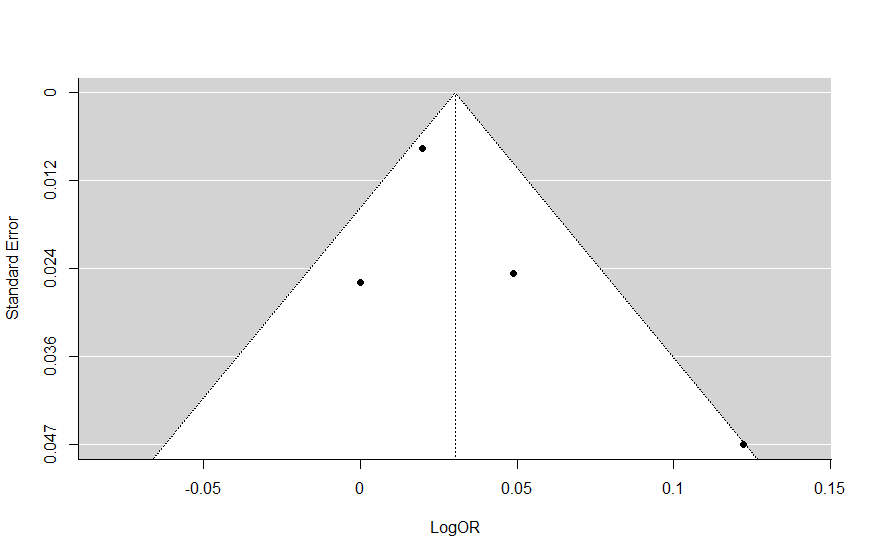
**

**Supplementary Table 3: Overview of reported associations in all included studies**

|  | **Absolute PHO** | | | **Relative PHO** | | | **PHO growth** | | | **OED** | | |
| --- | --- | --- | --- | --- | --- | --- | --- | --- | --- | --- | --- | --- |
|  | Poor | Neutral | Good | Poor | Neutral | Good | Poor | Neutral | Good | Poor | Neutral | Good |
| **Death at 1 month** | | | | | | | | | | | | |
| Gebel 2002 |  |  |  |  |  |  |  |  |  |  |  |  |
| Ozdinc 2016 |  |  |  |  |  |  |  |  |  |  |  |  |
| Gusdon 2020 |  |  |  |  |  |  |  |  |  |  |  |  |
| Pinho 2020 |  |  |  |  |  |  |  |  |  |  |  |  |
| Shirazian 2021 |  |  |  |  |  |  |  |  |  |  |  |  |
| **mRS score 3-6 at 3 months** | | | | | | | | | | | | |
| Gebel 2002 |  |  |  |  |  |  |  |  |  |  |  |  |
| Delgado 2006 |  |  |  |  |  |  |  |  |  |  |  |  |
| Gupta 2014 |  |  |  |  |  |  |  |  |  |  |  |  |
| Yang 2015 |  |  |  |  |  |  |  |  |  |  |  |  |
| Murthy 2016 |  |  |  |  |  |  |  |  |  |  |  |  |
| Rodriguez-Luna 2016 |  |  |  |  |  |  |  |  |  |  |  |  |
| Urday 2016 |  |  |  |  |  |  |  |  |  |  |  |  |
| Iglesias-Rey 2018 |  |  |  |  |  |  |  |  |  |  |  |  |
| Chen 2019 |  |  |  |  |  |  |  |  |  |  |  |  |
| Hurford 2019 |  |  |  |  |  |  |  |  |  |  |  |  |
| Huan 2021 |  |  |  |  |  |  |  |  |  |  |  |  |
| **mRS score 4-6 at 3 months** | | | | | | | | | | | | |
| Li 2013 |  |  |  |  |  |  |  |  |  |  |  |  |
| Volbers 2018 |  |  |  |  |  |  |  |  |  |  |  |  |
| Leasure 2019 |  |  |  |  |  |  |  |  |  |  |  |  |
| Lv 2021 |  |  |  |  |  |  |  |  |  |  |  |  |
| Nawabi 2021 |  |  |  |  |  |  |  |  |  |  |  |  |
| Shirazian 2021 |  |  |  |  |  |  |  |  |  |  |  |  |
| Ye 2021 |  |  |  |  |  |  |  |  |  |  |  |  |
| **Death at 3 months** | | | | | | | | | | | | |
| Alvarez-Sabin 2004 |  |  |  |  |  |  |  |  |  |  |  |  |
| Levine 2007 |  |  |  |  |  |  |  |  |  |  |  |  |
| Murthy 2016 |  |  |  |  |  |  |  |  |  |  |  |  |
| Rodriguez-Luna 2016 |  |  |  |  |  |  |  |  |  |  |  |  |
| **mRS score 3-6 at 6 months** | | | | | | | | | | | | |
| Tsai 2013 |  |  |  |  |  |  |  |  |  |  |  |  |
| **Death at 6 months** | | | | | | | | | | | | |
| Wu 2017 |  |  |  |  |  |  |  |  |  |  |  |  |
| **mRS score 3-6 at 12 months** | | | | | | | | | | | | |
| Loan 2021 |  |  |  |  |  |  |  |  |  |  |  |  |
| **Other: ‘worse mRS at 3 months’** | | | | | | | | | | | | |
| Sansing 2011 |  |  |  |  |  |  |  |  |  |  |  |  |

This table provides an overview of all available associations between PHO and outcome that are reported in the included studies. Both primary and secondary outcomes are presented.

= available association in this study. Green indicates a positive association between PHO and outcome, grey indicates a neutral association whereas red indicates an inverse association between PHO and outcome.

= not reported in this study.

**Supplementary Table 4: Odds ratios and their characteristics of the included studies**

| **Author (year)** | **Measure of PHO** | **Definition of poor outcome** | **Reported adjusted OR** | **Reported unadjusted OR** | **Adjusted for** |
| --- | --- | --- | --- | --- | --- |
| **mRS 3-6 at three months (figure 2)** | | | | | |
| Delgado *et al* (2006) | aPHO | mRS 3-6 at 3 months | NR | NR | - |
| Murthy *et al* (2016) | aPHO, rPHO, growth | mRS 3-6 at 3 months | 1.02 (1.01-1.04) | NR | Age, admission GCS, baseline ICH volume, lobar location, IVH, infratentorial location, warfarin use and time to baseline CT. |
| Rodriguez-Luna *et* (2016) | aPHO | mRS 3-6 at 3 months | NR | NR | - |
| Urday *et al* (2016) | aPHO | mRS 3-6 at 3 months | 1.00 (0.95-1.05) | 1.01 (0.99-1.03) | ICH score components (age, ICH volume, IVH volume, GCS) |
| Iglesias-Rey *et al* (2018) | aPHO | mRS 3-6 at 3 months | 1.05 (1.00-1.10) | NR | Based on univariable analysis:  Age, Body temperature, maximum temperature during first 24 hours, glucose level, C-reactive protein, sedimentation rate, ICH volume at admission, ICH volume at day 4-7, NIHSS score at admission, NIHSS score at 48 hours, early neurological deterioration, ICH aetiology |
| Chen *et al* (2019) | aPHO | mRS 3-6 at 3 months | NR | NR | - |
| Huan *et al* (2021) | aPHO, rPHO and OED | mRS 3-6 at 3 months | 1.13 (1.03-1.24) | NR | Age, admission GCS, ICH volume, ICH expansion, basal ganglia haemorrhage, IVH |
| **mRS 3-6 at any time of follow up** | | | | | |
| Tsai *et al* (2013) | rPHO | mRS 3-6 at 6 months | NR | 0.977 (0.431-2.214) | - |
| Loan *et al* (2020) | aPHO and OED | mRS 3-6 at 1 year | 0.92 (0.63-1.45) | NR | ICH volume, Age, IVH, ICH location, GCS score |
| **mRS 4-6 at 3 months (figure 3)** | | | | | |
| Li *et al* (2013) | aPHO | mRS 4-6 at 3 months | NR | NR | - |
| Volbers *et al* (2018) | Peak aPHO | mRS 0-3 at 3 months | 0.984 (0.973-0.994) | NR | IVH, NIHSS at admission, age, ICH volume by location (volume x location lobar vs basal ganglia) |
| Leasure *et al* (2019) | aPHO growth | mRS 4-6 at 3 months | 1.14 (0.93-1.40) | 1.43 (1.24–1.67) | Age, sex, admission GCS, admission ICH volume, presence of IVH, volume of haematoma expansion, and treatment group (blood pressure treatment versus conservative) |
| Lv *et al* (2021) | aPHO | mRS 4-6 at 3 months | NR | 1.12 (1.06-1.17) | - |
| Nawabi *et al* (2021) | aPHO | mRS 4-6 at 3 months | 1.015 (1.002-1.028) | NR | Sex, GCS, NIHSS, IVH and ICH location. |
| Shirazian *et al* (2021) | aPHO growth | mRS 4-6 at 3 months | 1.69 (1.2-2.37) | 8.0 (1.9–33.3) | Age, sex, race, baseline ICH volume, ICH location |
| Ye *et al* (2021) | aPHO growth | mRS 4-6 at 3 months | 1.05 (1.02-1.08) | NR | ICH volume |
| **Death at any time of follow up (figure 4)** | | | | | |
| Gebel *et al* (2002) | aPHO and rPHO | Death at 1 month | NR | NR | - |
| Alvarez-Sabin *et al* (2004) | aPHO | Death at 3 months | NR | NR | - |
| Levine *et al* (2007) | aPHO | Death at 3 months | per 100cc:  0.41 (0.19-0.91)  per 1cc:  0.991 (0.983-0.999) | NR | ICH volume, glucose, anti-platelet medication, warfarin |
| Murthy *et al* (2016) | aPHO, rPHO, growth | Death at 3 months | 1.02 (0.98-1.04) | NR | Age, admission GCS, baseline ICH volume, lobar location, infratentorial location, IVH, warfarin use and time to baseline CT. |
| Ozdinic *et al* (2016) | aPHO | Death at 30 days | NR | NR | - |
| Rodriguez-Luna *et al* (2016) | aPHO | Death at 3 months | NR | NR | - |
| Wu *et al* (2017) | aPHO and OED | Death at 6 months | 1.02 (1.01-1.02) | NR | Age, baseline NIHSS score, baseline GCS score, prestroke warfarin use, baseline ICH volume, and ventricular extension. |
| Gusdon *et al* (2020) | aPHO | Death at 1 month | 1.07 (1.04-1.09) | NR | ICH volume, platelet transfusion, chemotherapy, radiation therapy, ICH location |
| Pinho *et al* (2020) | aPHO and rPHO | Death at 1 month | 1.00 (0.98-1.02) | 1.03 (1.02–1.04) | Age, sex, GCS score, blood glucose, haemoglobin, neutrophil/ lymphocyte ratio, INR, red cell distribution, IVH, ICH volume |
| Shirazian *et al* (2021) | aPHO growth | Death at 1 month | 2.78 (1.40–5.54) | 3.80; (1.20–11.98) | Age, sex, race, baseline ICH volume, location of haematoma |
| **PHO growth and any form of functional outcome at any time of follow up (figure 5)** | | | | | |
| Yang *et al* (2015) | aPHO growth | mRS 3-6 at 3 months | 1.03 (1.00-1.06) | 1.04 (1.02-1.06) | Age, sex, country of residence, prior ICH, ischemic stroke, acute coronary syndrome, diabetes mellitus, hypertension, use of an antithrombotic and lipid lowering agent, time to baseline CT, admission systolic blood pressure, admission glucose, high (≥14) NIHSS score, lobar haematoma, IVH, baseline ICH volume, 24 hour ICH growth, mannitol treatment, randomized treatment and trial |
| Murthy *et al* (2016) | aPHO growth | mRS 3-6 at 3 months | 1.67 (1.28-2.39) | NR | Age, admission GCS, baseline ICH volume, lobar location, infratentorial location, IVH, warfarin use and time to baseline CT. |
| Hurford *et al* (2019) | OED growth | mRS 3-6 at 3 months | 1.96 (1.00-3.83) | NR | Age, sex, diabetes, antiplatelet therapy, anticoagulation, statin use, lobar haemorrhage, ICH volume at 72h, IVH at 72h |
| Leasure *et al* (2019) | aPHO growth | mRS 4-6 at 3 months | 1.14 (0.93-1.40) | 1.43 (1.24–1.67) | Age, sex, admission GCS, admission ICH volume, IVH, volume of haematoma expansion, and treatment group (blood pressure treatment versus conservative) |
| Gusdon *et al* (2020) | aPHO growth | Death at 1 month | 1.05 (1.01-1.10) | NR | ICH volume, platelet transfusion, chemotherapy, radiation therapy, ICH location |
| Lv *et al* (2021) | aPHO growth | mRS 4-6 at 3 months | 4.25 (1.70-10.60) | NR | Age, admission systolic blood pressure, GCS score, baseline ICH volume, the presence of IVH, and time from onset to CT |
| Shirazian *et al* (2021) | aPHO growth | mRS 4-6 at 3 months | 1.69 (1.2-2.37) | 8.0 (1.9–33.3) | Age, sex, race, baseline ICH volume, ICH location |
| Ye *et al* (2021) | aPHO growth | mRS 4-6 at 3 months | 1.05 (1.02-1.08) | NR | ICH volume |
| **Other studies** | | | | | |
| Sansing et al (2011) | aPHO | Worse mRS at 3 months | 1.04 (1.02-1.05) | NR | Age, baseline ICH volume, infratentorial location, IVH, presenting GCS score. |
| Gupta et al (2014) | rPHO | mRS 3-6 at 3 months | 0 (0.00-0.008) | NR | unclear |

Abbreviations: aPHO = absolute perihaematomal oedema; CT = computed tomography scan; GCS = Glasgow Coma Scale; ICH = intracerebral haemorrhage; INR = international normalised ratio; IVH = intraventricular haemorrhage; mRS = modified Rankin scale score; NIHSS = National Institute of Stroke Scale; NR = not reported; OED = oedema extension distance; OR = odds ratio; PHO = perihaematomal oedema; rPHO = relative perihaematomal oedema.

**Supplementary figure 2:**

**Estimates of the association between perihaematomal oedema and death at any time of follow up.**


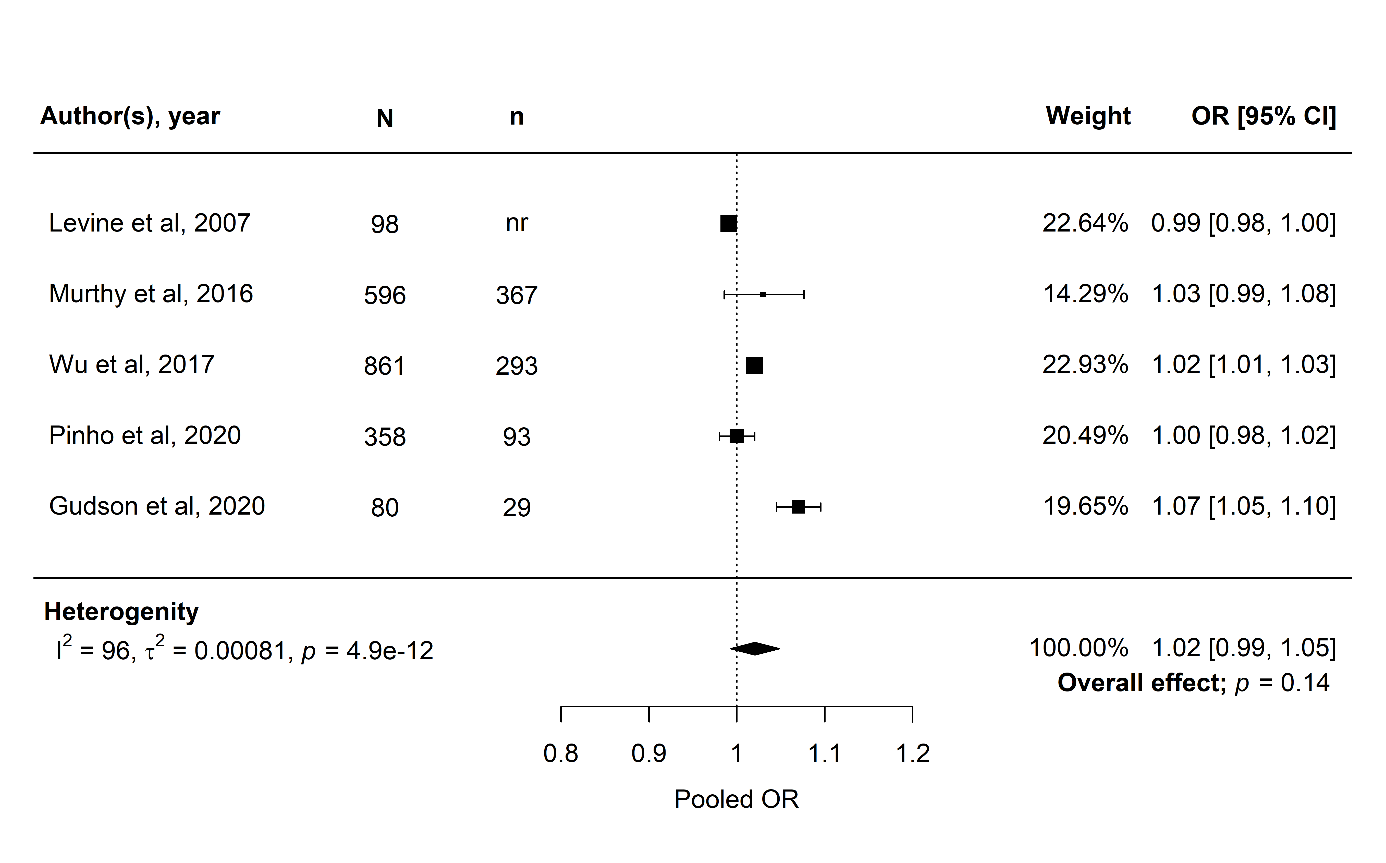
N = total participants; n= participants with poor outcome; OR = odds ratio; 95% CI = 95% confidence interval. All studies reported an OR adjusted for at least ICH volume. Murthy (2016) adjusted for an additional seven factors, Wu (2017) for an additional five, Gusdon (2020) for an additional four and Pinho (2020) for an additional ten factors (Supplementary Table 4).
